# Novel Antimicrobials from Uncultured Bacteria Acting against Mycobacterium tuberculosis

**DOI:** 10.1128/mBio.01516-20

**Published:** 2020-08-04

**Authors:** Jeffrey Quigley, Aaron Peoples, Asel Sarybaeva, Dallas Hughes, Meghan Ghiglieri, Catherine Achorn, Alysha Desrosiers, Cintia Felix, Libang Liang, Stephanie Malveira, William Millett, Anthony Nitti, Baldwin Tran, Ashley Zullo, Clemens Anklin, Amy Spoering, Losee Lucy Ling, Kim Lewis

**Affiliations:** a Antimicrobial Discovery Center, Department of Biology, Northeastern University, Boston, Massachusetts, USA; b NovoBiotic Pharmaceuticals, LLC, Cambridge, Massachusetts, USA; c Bruker Biospin Corporation, Billerica, Massachusetts, USA; New York University School of Medicine

**Keywords:** drug discovery, *Mycobacterium tuberculosis*, natural product discovery, nontuberculous mycobacteria, Sec translocation, antibiotic, antimicrobial

## Abstract

Mycobacterium tuberculosis, which causes tuberculosis (TB), is estimated to infect one-third of the world’s population. The overall burden and the emergence of drug-resistant strains of Mycobacterium tuberculosis underscore the need for new therapeutic options against this important human pathogen. Our recent work demonstrated the success of natural product discovery in identifying novel compounds with efficacy against Mycobacterium tuberculosis. Here, we improve on these methods by combining improved isolation and Mycobacterium tuberculosis selective screening to identify three new anti-TB compounds: streptomycobactin, kitamycobactin, and amycobactin. We were unable to obtain mutants resistant to streptomycobactin, and its target remains to be elucidated. We identify the target of kitamycobactin to be the mycobacterial ClpP1P2C1 protease and confirm that kitamycobactin is an analog of the previously identified compound lassomycin. Further, we identify the target of amycobactin to be the essential protein secretion pore SecY. We show further that amycobactin inhibits protein secretion via the SecY translocon. Importantly, this inhibition is bactericidal to nonreplicating Mycobacterium tuberculosis. This is the first compound, to our knowledge, that targets the Sec protein secretion machinery in Mycobacterium tuberculosis. This work underscores the ability of natural product discovery to deliver not only new compounds with activity against Mycobacterium tuberculosis but also compounds with novel targets.

## Abstract (precis)

Decreasing discovery rates and increasing resistance have underscored the need for novel therapeutic options to treat Mycobacterium tuberculosis infection. Here, we screen extracts from previously uncultured soil microbes for specific activity against Mycobacterium tuberculosis, identifying three novel compounds. We further define the mechanism of action of one compound, amycobactin, and demonstrate that it inhibits protein secretion through the Sec translocation machinery.

## INTRODUCTION

Mycobacterium tuberculosis is the leading cause of death due to a single infectious agent worldwide (1). The treatment currently recommended for infection with drug-susceptible M. tuberculosis is a 2-month intensive chemotherapy regimen of the four first-line antibiotics rifampin, isoniazid, pyrazinamide, and ethambutol followed by a 4- to 6-month continuation phase consisting of rifampin and isoniazid (1). Poor patient compliance due to prolonged treatment and toxic side effects of these compounds has led to the emergence of multidrug-resistant and extensively drug-resistant strains of M. tuberculosis (2, 3), underscoring the need for new treatment options.

Most antibiotics, beginning with penicillin and streptomycin, are natural products or their derivatives (4, 5). However, the inability to culture most bacteria under laboratory conditions, as well as the continual rediscovery of known compounds, led to diminishing returns, and natural product screening efforts were largely abandoned by the 1960s (6). Improved methods of growing previously uncultured bacteria now provide access to untapped biological and chemical diversity. A previously uncultured bacterium, Eleftheria terrae, produces teixobactin, a novel cell wall-acting inhibitor (7). We also introduced a selective screening approach that is aimed at eliminating the large background of toxic and known compounds. Screening of extracts from uncultured bacteria against M. tuberculosis, and counterscreening against Staphylococcus aureus, led to the discovery of lassomycin, produced by a *Lentzea* sp., which targets the C1 subunit of the essential P1P2C1 protease of mycobacteria (8).

Here, we report the identification of three novel antimicrobials from previously uncultured bacteria with selective activity against M. tuberculosis. Streptomycobactin is a cationic depsipeptide, kitamycobactin is a lasso peptide that requires native C1 for activity, and amycobactin is a novel antimicrobial that targets the SecY protein of the mycobacterial secretion system.

## RESULTS

### Identification of M. tuberculosis selective compounds.

**FIG 1:**
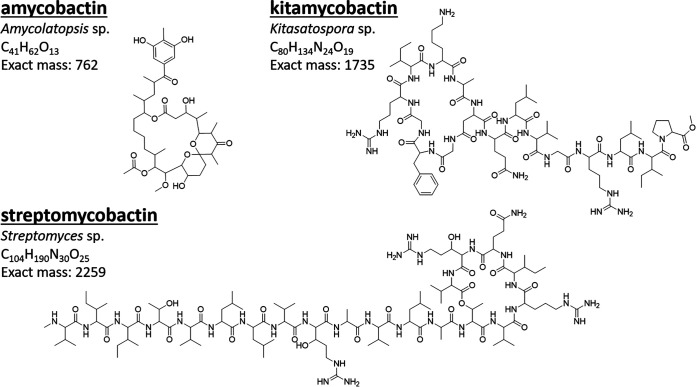
Structures of compounds. Shown are the chemical structures of compounds identified in screening. The genera of the producing organisms and the exact mass in Daltons of each compound are indicated.

Extracts prepared from the fermentation broth of 10,241 previously uncultured bacteria were screened for the ability to inhibit the growth of M. tuberculosis and S. aureus. Extracts with activity against M. tuberculosis but not S. aureus were selected for follow-up studies. From this screen, four extracts were selected based on reproducibility of activity. The extracts were fractionated by high-performance liquid chromatography (HPLC) until a single active fraction was identified by bioactivity-guided purification. The structures of the four compounds were determined by nuclear magnetic resonance (NMR) analysis. Three compounds were determined to be novel, since they did not have matches in AntiBase or SciFinder. The fourth was determined to be the previously identified compound marfomycin D, purified from a deep-sea *Streptomyces* species. However, its activity against mycobacteria had not been reported (9). The target of marfomycin D is unknown. We named the novel compounds amycobactin, kitamycobactin, and streptomycobactin (Fig. 1). The compounds were named by combining the name of the genus of the producing species with the mycobactin suffix to indicate activity against mycobacteria. The structures of amycobactin (Table S1, Fig. S1, Fig. S2, and Text S1) and kitamycobactin (Table S2, Fig. S3, and Text S2) were assigned by ^1^H, ^13^C, correlation spectroscopy (COSY), total correlation spectroscopy (TOCSY), ^1^H-^13^C/^15^N heteronuclear single quantum coherence (HSQC), ^1^H-^13^C heteronuclear multiple-bond correlation (HMBC), and nuclear Overhauser effect spectroscopy (NOESY)/rotating-frame nuclear Overhauser effect spectroscopy (ROESY) experiments. For elucidation of the structure of streptomycobactin, additional triple-resonance experiments on a universally ^13^C- and ^15^N-labeled sample were used. ^13^C chemical shifts of the peptidyl backbone were mapped using HNCACB and HNCOCACB experiments, while the side-chain ^13^C and ^1^H chemical shifts were assigned using (H)CCCONH and H(CCCO)NH experiments, respectively (Table S3, Fig. S4, and Text S3).

10.1128/mBio.01516-20.1 FIG S1 Key ^1^H-^1^H COSY/TOCSY and ^1^H-^13^C HMBC correlations of amycobactin. COSY correlations of H18–H19, H12–H13, and H10–H11 are unable to be assigned unambiguously due to proton signal overlap. The full structure was assigned by 1D ^1^H and ^13^C chemical shifts and 2D correlations leaving two open methylene groups at C-18 and C-19. The carbon and proton chemical shifts at these two positions eliminate any possibility of their being adjacent to any heteroatoms. Therefore, C-18 and C-19 must be connected by a single bond. Download FIG S1, TIF file, 0.1 MB. Copyright © 2020 Quigley et al. 2020 Quigley et al. This content is distributed under the terms of the Creative Commons Attribution 4.0 International license.

10.1128/mBio.01516-20.2 FIG S2 Key NMR spectra of amycobactin. (A) ^1^H; (B) ^13^C DEPT135; (C) ^1^H-^1^H COSY; (D) ^1^H-^13^C HSQC; (E) ^1^H-^13^C HMBC; (F) NOESY. Download FIG S2, TIF file, 0.3 MB. Copyright © 2020 Quigley et al. 2020 Quigley et al. This content is distributed under the terms of the Creative Commons Attribution 4.0 International license.

10.1128/mBio.01516-20.3 FIG S3 Key NMR spectra of streptomycobactin. (A) ^1^H; (B) ^13^C; (C) ^1^H-^1^H COSY; (D) ^1^H-^13^C HSQC; (E) ^1^H-^13^C HMBC; (F) ^1^H-^15^N HSQC; (G) HNCACB; (H) HN(CO)CACB; (I) CCCONH; (J) ^15^N TOCSY-HSQC. Download FIG S3, TIF file, 0.5 MB. Copyright © 2020 Quigley et al. 2020 Quigley et al. This content is distributed under the terms of the Creative Commons Attribution 4.0 International license.

10.1128/mBio.01516-20.4 FIG S4 Key NMR spectra of kitamycobactin. (A) ^1^H; (B) ^13^C; (C) ^1^H-^1^H COSY; (D) ^1^H-^13^C HSQC; (E) ^1^H-^13^C HMBC; (F) ^1^H-^1^H TOCSY; (G) ^1^H-^15^N HSQC; (H) ^1^H-^1^H ROESY. Download FIG S4, TIF file, 0.5 MB. Copyright © 2020 Quigley et al. 2020 Quigley et al. This content is distributed under the terms of the Creative Commons Attribution 4.0 International license.

10.1128/mBio.01516-20.5 TABLE S1 ^1^H and ^13^C NMR data of amycobactin (500 and 125 MHz in DMSO-*d_6_*, δ in ppm). Download Table S1, DOCX file, 0.02 MB. Copyright © 2020 Quigley et al. 2020 Quigley et al. This content is distributed under the terms of the Creative Commons Attribution 4.0 International license.

10.1128/mBio.01516-20.6 TABLE S2 ^1^H, ^13^C, and ^15^N NMR data of streptomycobactin (700, 175, and 70 MHz in DMSO-*d_6_*, δ in ppm). Download Table S2, DOCX file, 0.02 MB. Copyright © 2020 Quigley et al. 2020 Quigley et al. This content is distributed under the terms of the Creative Commons Attribution 4.0 International license.

10.1128/mBio.01516-20.7 TABLE S3 ^1^H, ^13^C, and ^15^N NMR data of kitamycobactin (500, 125, and 50 MHz in DMSO-*d_6_*, δ in ppm). Download Table S3, DOCX file, 0.01 MB. Copyright © 2020 Quigley et al. 2020 Quigley et al. This content is distributed under the terms of the Creative Commons Attribution 4.0 International license.

10.1128/mBio.01516-20.8 TEXT S1 Detailed data interpretation of structural determination of amycobactin. Download Text S1, DOCX file, 0.01 MB. Copyright © 2020 Quigley et al. 2020 Quigley et al. This content is distributed under the terms of the Creative Commons Attribution 4.0 International license.

10.1128/mBio.01516-20.9 TEXT S2 Detailed data interpretation of structural determination of streptomycobactin. Download Text S2, DOCX file, 0.01 MB. Copyright © 2020 Quigley et al. 2020 Quigley et al. This content is distributed under the terms of the Creative Commons Attribution 4.0 International license.

10.1128/mBio.01516-20.10 TEXT S3 Detailed data interpretation of structural determination of kitamycobactin. Download Text S3, DOCX file, 0.01 MB. Copyright © 2020 Quigley et al. 2020 Quigley et al. This content is distributed under the terms of the Creative Commons Attribution 4.0 International license.

Amycobactin is produced by an *Amycolatopsis* sp., has an exact mass of 762 Da, and is the smallest of the compounds identified. It has an unusual structure, featuring a ketal moiety within a macrolactone backbone. Kitamycobactin and streptomycobactin are semicyclic peptides with exact masses of 2,259 and 1,735 Da, respectively. Streptomycobactin is a 20-amino-acid peptide (VIITVLLVRAVLATVRIERV) produced by a *Streptomyces* sp. Kitamycobactin, produced by a *Kitasatospora* sp., is a lasso peptide (GFGRIKADELVGRLIP) and belongs to the same class as lassomycin. Antimicrobials have been identified from these genera previously. However, our *in situ* cultivation method utilizing the iChip increases recovery 50-fold (10). This means that, using our methods, the probability that an isolate represents a conventional cultivable species is 1/50; for the three isolates described here, the probability is 8 × 10^−6^. This makes it highly unlikely that the producing species have been cultivated previously.

### Compound bioactivity.

The compounds were tested for inhibition of the growth of M. tuberculosis, as well as inhibition of the growth of other mycobacterial pathogens and some gut symbionts (Table 1). Rifampin was used as a positive control in all MIC testing against M. tuberculosis H37Rv (MIC, 0.5 μg/ml) and mc^2^6020 (MIC, 0.125 μg/ml). We included susceptibility data for marfomycin D as well, since this compound had not been tested against mycobacteria (9). Streptomycobactin and kitamycobactin were the most potent compounds, with MICs of 0.03 μg/ml and 0.06 μg/ml, respectively. While efforts to identify new antimycobacterial compounds focus mainly on M. tuberculosis, there has been increasing interest recently in nontuberculous mycobacteria (NTM). The NTM family of organisms encompasses >150 different species of opportunistic pathogens that cause various diseases ranging from pulmonary infections similar to tuberculosis to soft-tissue and skin infections (11). Increasing rates of drug resistance have led to a need for new therapeutic options to treat NTM infections. With this in mind, we tested the compounds against the major NTM pathogens: Mycobacterium avium, Mycobacterium abscessus, and Mycobacterium paratuberculosis. Clarithromycin was used as a positive control, with MICs of 0.5 μg/ml, 0.25 μg/ml, and 0.25 to 0.125 μg/ml against M. avium, M. abscessus, and M. paratuberculosis, respectively. Overall, the compounds proved to be effective against the NTM species at a level similar to their effectiveness against M. tuberculosis (Table 1).

**TABLE 1:** Bioactivity of compounds

Organism or cell type	MIC (μg/ml) or TC_50_^*a*^			
	Amycobactin	Streptomycobactin	Kitamycobactin	Marfomycin D
Pathogens				
M. tuberculosis mc^2^6020	4–8	0.03	0.06	1
M. tuberculosis H37Rv	4–8	0.03	0.06	0.03
M. avium	4	<0.1	<0.1	<0.1
M. abscessus	16–32	0.5	1	>64
M. paratuberculosis	2–4	0.1–0.25	<0.1	4
S. aureus	>64	4	>128	>64
Gut symbionts				
Bacteroides fragilis	32	32	>64	>64
Lactobacillus reuteri	32	2	8	>64
Mammalian cells				
HepG2	16–32	16–32	100	16–32
NIH/3T3	16–32	16–32	>100	>50

a Values are MICs for bacteria and TC_50_ for mammalian cells.

The detrimental effects of antibiotic treatment on the gut microbiome are a well-recognized phenomenon (12). With this in mind, we tested all the compounds against the gut commensal bacteria Bacteroides fragilis and Lactobacillus reuteri (Table 1). The limited activity of the compounds against commensal bacteria confirms their selectivity against M. tuberculosis. We also determined cytotoxicity against the liver cell line HepG2 and the mouse embryonic fibroblast cell line NIH/3T3. Kitamycobactin had no activity at the highest concentration tested, 100 μg/ml. Amycobactin and streptomycobactin both had 50% toxic concentrations (TC_50_) of 16 to 32 μg/ml against HepG2 and NIH/3T3 cells. Given the streptomycobactin MIC of 0.03 μg/ml against M. tuberculosis, this creates a significant therapeutic window. However, the therapeutic window for amycobactin is considerably smaller, given its MIC of 4 to 8 μg/ml against M. tuberculosis. Overall, the compounds displayed good selectivity against M. tuberculosis and mycobacteria, with limited cytotoxicity.

**FIG 2:**
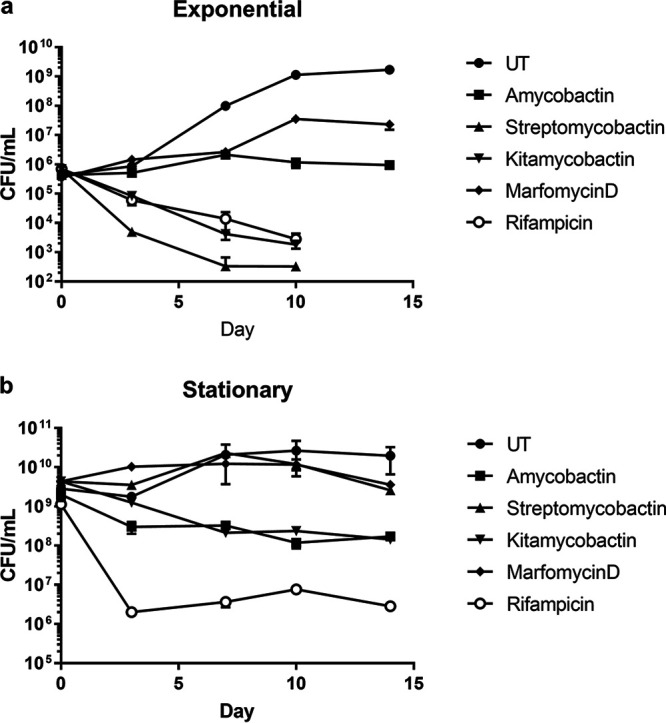
Compound activity against exponentially growing and stationary-phase M. tuberculosis cultures. (a) M. tuberculosis was grown to mid-log phase and diluted to an OD_600_ of 0.003. The cultures were either left untreated (UT) or treated with each compound at 4× MIC (amycobactin) or 10× MIC (streptomycobactin, kitamycobactin, and marfomycin D). Samples were taken at the indicated time points and plated for CFU counting. (b) Stationary-phase cultures of M. tuberculosis were either left untreated or treated with each compound at 4× MIC (amycobactin) or 10× MIC (streptomycobactin, kitamycobactin, and marfomycin D). Samples were plated for CFU counting at the indicated time points. Data represent the results of two replicates and are displayed as means ± standard errors of the means.

The killing kinetics were determined for all four compounds against M. tuberculosis strain mc^2^6020 in both the exponential- and stationary-growth phases. Amycobactin was bacteriostatic in exponential phase (Fig. 2a) but, interestingly, was moderately bactericidal (<1-log killing) in stationary phase (Fig. 2b). Streptomycobactin and kitamycobactin were both bactericidal to exponentially growing cultures (Fig. 2a), while kitamycobactin displayed bactericidal activity against stationary-phase cultures (Fig. 2b). Marfomycin D was bacteriostatic against exponential-phase cultures until day 7, after which efficacy was lost and the cultures began to grow (Fig. 2a). The ability of M. tuberculosis to enter nonreplicating or slowly replicating states, such as stationary phase, is considered a primary reason underlying treatment failure (13, 14). Compounds with effectiveness against M. tuberculosis in these nonreplicating or slowly replicating states, such as amycobactin and kitamycobactin, are essential to reducing treatment duration and increasing treatment success.

### Target identification.

We next sought to generate mutants in order to identify the targets of the compounds. Using single-step or sequential selection, we were unable to generate mutants against streptomycobactin. However, given the sensitivity of M. tuberculosis to this compound and the large therapeutic window (Table 1), continued efforts to understand the mechanism of action are warranted. As mentioned above, the general architecture of kitamycobactin resembles that of the M. tuberculosis-selective compound lassomycin (8). We therefore tested a ClpC1 Q17R mutant resistant to lassomycin (8) and found that it was resistant to kitamycobactin as well. Kitamycobactin had a MIC of 4 μg/ml against the ClpC1 Q17R mutant, while its MIC against the parental strain, mc^2^6020, is 0.06 μg/ml (Table 1). This suggests that kitamycobactin acts against ClpP1P2C1, the essential protease of mycobacteria. Action against this target explains the selectivity of kitamycobactin against mycobacteria. The independent evolution of two compounds, lassomycin and kitamycobactin, that interfere with the same essential target is noteworthy. This would suggest that the ClpP1P2C1 protease is an attractive target for the inhibition of mycobacteria by soil microbes.

**FIG 3:**
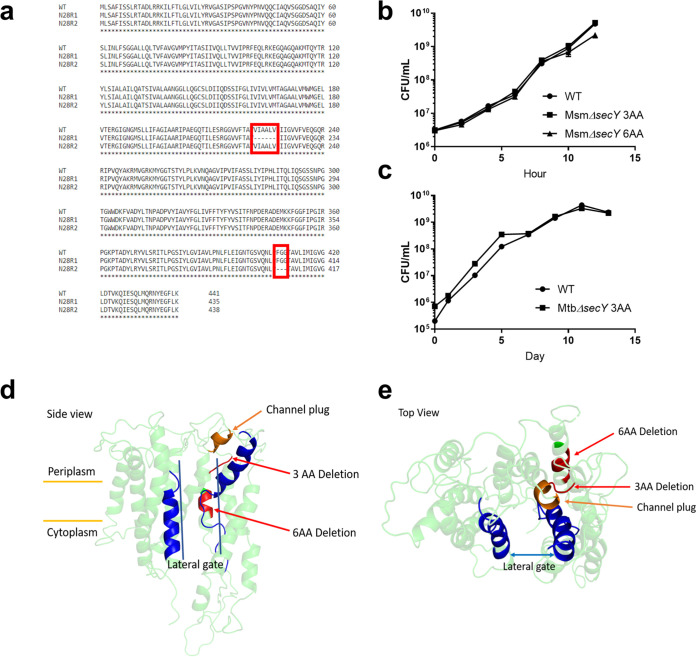
Analysis of amycobactin mutants in M. smegmatis and M. tuberculosis. (a) Alignment of protein sequences from WT M. smegmatis SecY and amycobactin mutants N28R1 and N28R2. The deletions in each mutant are boxed in red. (b) Growth curves of WT M. smegmatis and mutants containing targeted 3- and 6-amino-acid deletions in *secY* conferring resistance to amycobactin. (c) Growth curves of WT M. tuberculosis and a mutant containing a targeted 3-amino-acid deletion in *secY* conferring resistance to amycobactin. Data in panels b and c represent the results of two independent experiments and are displayed as means ± standard errors of the means. (d and e) Side view (d) and top view (e) of the predicted crystal structure of M. tuberculosis SecY, with the amycobactin resistance-conferring mutations shown in red. For reference, helices 2 and 7, which together form the lateral gate of SecY, are shown in blue. The plug restricting secretion through the central channel of SecY is shown in orange.

Attempts to obtain mutants of M. tuberculosis resistant to amycobactin failed, but we were able to generate two independent mutants (designated N28R1 and N28R2) in the related species Mycobacterium smegmatis. The amycobactin MIC against wild-type (WT) M. smegmatis was 1.5 μg/ml, while the MICs against N28R1 and N28R2 were 12.5 μg/ml and >100 μg/ml, respectively. Sequencing revealed that both mutants contained in-frame deletions in the general protein secretion translocase SecY (MSMEG1483), which, together with SecE and SecA, forms the core of the Sec protein translocation machinery (15, 16). Sec secretion is essential in all bacteria and is responsible for the translocation of a majority of secreted and integral membrane proteins (17, 18). N28R1 contained an 18-bp deletion in the *secY* gene, resulting in the deletion of amino acids 222 to 227 (VIAALV). N28R2 contained a 9-bp deletion in *secY*, resulting in the deletion of amino acids 407 to 409 (FGG) (Fig. 3a). Given the novelty of SecY as a putative target, we pursued the mechanism of action further.

Deletions corresponding to those in N28R1 and N28R2 were made in a clean background of WT M. smegmatis using site-directed recombineering (19, 20). The deletions were confirmed by sequencing, and the mutants were renamed Msm*ΔsecY* 6AA and Msm*ΔsecY* 3AA. The MIC for Msm*ΔsecY* 6AA was 12.5 μg/ml, and the MIC for Msm*ΔsecY* 3AA was >100 μg/ml, confirming that these deletions confer resistance to amycobactin. Both mutants grew as well as the wild type, indicating no apparent fitness cost of the *secY* deletions (Fig. 3b). We next sought to re-create these mutations in M. tuberculosis in order to confirm a conserved target in both species. The sequences of the SecY proteins in M. smegmatis and M. tuberculosis are very similar, including both the 3- and 6-amino-acid deletion regions. Site-directed mutagenesis targeting M. tuberculosis *secY* (Rv0732) was used to re-create these deletions in M. tuberculosis. The deletion of amino acids 407 to 409 was successful; it was confirmed by sequencing, and the mutant was named Mtb*ΔsecY* 3AA. We were unsuccessful in deleting amino acids 222 to 227 in M. tuberculosis despite several attempts. Mtb*ΔsecY* 3AA grew similarly to WT M. tuberculosis (Fig. 3c) and had a MIC of >100 μg/ml. Such large deletions are rare and may impose significant restrictions on protein function. Phyre2 (21) was used to predict the structure of M. tuberculosis SecY with 100% confidence to the highest-scoring template (PDB ID 2ZQP). The 6- and 3-amino-acid deletions were mapped to the predicted structure and are shown in red in Fig. 3d and e. The secretion of peptides through SecY in Escherichia coli is restricted by a hydrophobic core ring and a channel plug requiring ATP hydrolysis via SecA for secretion (22). The predicted channel plug is shown in orange in Fig. 3d and e. The lateral gate of SecY, formed by helices 2 and 7 (23), is shown in blue. The SecY mutations conferring resistance to amycobactin are near the channel plug and lie within the hydrophobic core ring (Fig. 3d and e). This suggests that amycobactin may interfere with the normal functioning of these structural components of SecY. Considering that all bacteria contain *secY*, it is unclear why amycobactin is selective against mycobacteria. The 3-amino-acid deletion conferring complete resistance (amino acids 407 to 409) is conserved in both E. coli and S. aureus. However, while M. tuberculosis SecY shares 88% identity with M. smegmatis SecY, it shares only 41% and 40% identity with the E. coli and S. aureus SecY proteins, respectively. It is likely this overall dissimilarity that accounts for the selectivity against mycobacteria.

**FIG 4:**
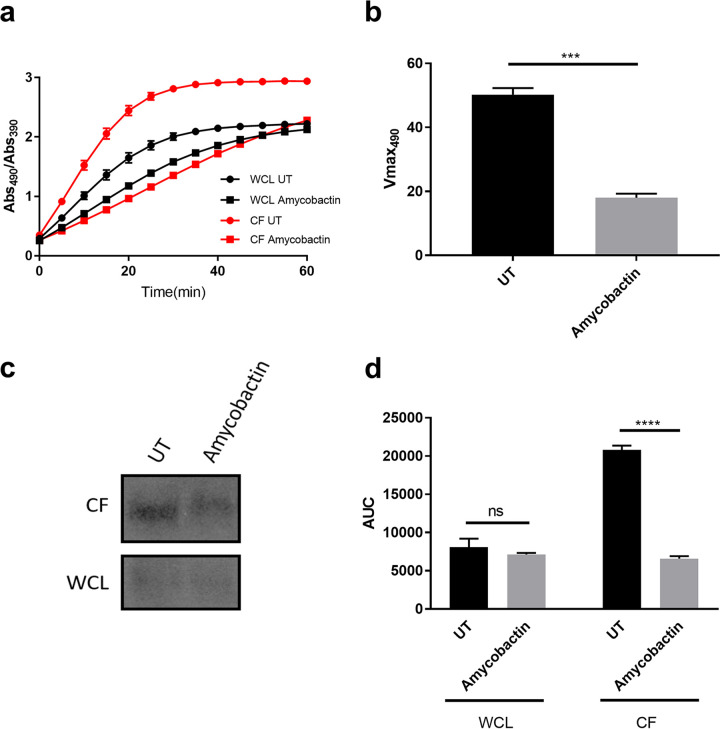
Amycobactin inhibits protein secretion through the Sec translocon. (a) The M. smegmatis ′BlaTEM-1 reporter strain was used to monitor the presence of β-lactamase in the culture supernatant (CF) (red curves) and whole-cell lysate (WCL) (black curves), either untreated (UT) (circles) or after treatment with amycobactin (squares). β-Lactamase was monitored by using the cleavage of the chromogenic β-lactamase substrate nitrocefin and monitoring the absorbance at 490 nm and 390 nm every 5 min for 60 min. The data, displayed as the ratio of absorbance at 490 nm (cleaved product) to absorbance at 390 nm (uncleaved nitrocefin), represent the results of three independent experiments. (b) Culture filtrate samples were analyzed for the maximum change in absorbance at 490 nm (*V*_*max*490_) over the course of the 60-minute experiment for which results are shown in panel a. (c) Representative Western blot analysis of β-lactamase protein in the CF and WCL of the untreated or amycobactin-treated M. smegmatis ′BlaTEM-1 reporter strain. (d) Densitometry analysis, using ImageJ software, of the Western blots of WCL and CF from untreated and amycobactin-treated cultures. AUC, area under the curve. Error bars display standard errors of the means. Significance was determined by Student’s *t* test (b) or one-way analysis of variance (d). ***, *P* ≤ 0.001; ****, *P* ≤ 0.0001; ns, not significant. Data represent the results of three independent experiments.

We next sought to determine whether amycobactin inhibited protein secretion by the Sec translocase. For this purpose, we turned to a strain of M. smegmatis engineered to secrete the E. coli ′BlaTEM-1 β-lactamase via the Sec translocation machinery (24). This strain was constructed by the fusion of E. coli ′BlaTEM-1 to the peptide secretion signal from MPT63, a protein known to be secreted through Sec. Additionally, the endogenous copy of β-lactamase in M. smegmatis was deleted (24). By using this strain, we can easily monitor protein secretion through the Sec translocase by monitoring the activity of the exported β-lactamase. A microtiter assay was used to determine the amount of β-lactamase in the culture filtrate (CF) and whole-cell lysate (WCL) by monitoring the hydrolysis of the chromogenic substrate nitrocefin over time. The cleavage of nitrocefin by β-lactamase results in a shift in the absorbance of nitrocefin from 490 nm to 390 nm (25, 26). Here, we monitored the hydrolysis of nitrocefin by the ratio of the absorbance at 490 nm to the absorbance at 390 nm. As shown in Fig. 4a, treatment of the reporter strain with amycobactin resulted in a lower level of nitrocefin hydrolysis in the culture filtrate of the amycobactin-treated sample than in the untreated control, indicating a decrease in the amount of β-lactamase present. Additionally, the maximum rate of the change in absorbance at 490 nm (*V*_*max*490_) was significantly greater in the untreated sample than in the amycobactin-treated sample CF (Fig. 4b), indicating a decrease in the amount of β-lactamase present after amycobactin treatment. Importantly, amycobactin treatment only moderately decreased the hydrolysis of nitrocefin in the WCL (Fig. 4a).

To confirm that amycobactin treatment inhibits the secretion of β-lactamase by the reporter strain, we used Western blotting to directly quantify the amounts of β-lactamase in the CF and WCL. Treatment of the reporter strain with amycobactin resulted in a significant decrease in the amount of β-lactamase in the CF from that in untreated cells (Fig. 4c and d). Importantly, the amount of β-lactamase in the WCL was unchanged after treatment (Fig. 4c). Taken together, these findings confirm that amycobactin acts to inhibit protein secretion through the Sec translocation machinery.

## DISCUSSION

Recent work has reiterated the power of natural product discovery to deliver promising new antimicrobials (27–29). Here, we turned our attention to previously uncultured microbes as a novel source of antimycobacterials. Coupling this method with differential screening for compounds acting selectively against M. tuberculosis, we identified three new antimicrobials with activity against mycobacteria and identified the targets for two of them, amycobactin and kitamycobactin.

Amycobactin directly targets protein secretion by the Sec translocation machinery and, to our knowledge, represents the first natural product targeting the protein secretion machinery. Amycobactin is bacteriostatic against exponentially growing M. tuberculosis but bactericidal against stationary-phase M. tuberculosis, which is puzzling, since antibiotics are generally more effective against actively growing cells. While amycobactin likely blocks the secretion of vital proteins in both states, its bactericidal activity in stationary phase may be due to the phenomenon of Sec translocon “jamming” (30). When secretion through the Sec translocation machinery fails, the SecY translocase becomes “jammed” with a linear peptide. The cell’s response is to target the entire apparatus for degradation by cellular proteases. This process itself can be fatal to the cell (30). Exponentially growing cells can easily replace the Sec machinery that was degraded. However, the decreased metabolic state of stationary-phase cells may limit the production of new Sec secretion components, resulting in killing by amycobactin.

The essential components of the Sec translocation machinery in M. tuberculosis and M. smegmatis are SecY, SecE, and SecA (17). SecE forms a “clamp” near the lateral gate of SecY to stabilize the structure (22). SecA provides the recognition of secreted proteins as well as the energy for secretion by hydrolyzing ATP (31). Amycobactin may act to disrupt the interaction of SecY with SecE or SecA, inhibiting secretion. Alternatively, amycobactin may act directly on SecY. Secretion through SecY is restricted by a hydrophobic core ring and a channel plug; the latter is displaced during secretion (22). Amycobactin may act to restrict the displacement of the channel plug in WT SecY, resulting in blocked secretion. Deletion of amino acids 407 to 409 may alleviate this blockage by providing more flexibility in the channel plug.

The high MIC and narrow therapeutic window of amycobactin suggest that this is an early lead that could be further optimized. Its inhibition of protein secretion also makes it an attractive tool for study of the Sec translocation machinery. The essentiality of the Sec secretion machinery makes studying its contribution to virulence challenging. Considering that several virulence-associated proteins are secreted via the Sec machinery (17, 32), a tool for studying this aspect of M. tuberculosis physiology would be invaluable. Taking these considerations together, this work underscores the power of natural product discovery to deliver promising new antibiotics with novel mechanisms of action against M. tuberculosis.

## MATERIALS AND METHODS

### Bacterial strains and growth conditions.

The M. tuberculosis strains used were H37Rv and H37Rv mc^2^6020 where noted. M. smegmatis strain mc^2^155 was used for all M. smegmatis work. M. avium strain ATCC 700898, M. abscessus strain ATCC 19977, and M. paratuberculosis strain ATCC 43544 were used for antibiotic susceptibility. All mycobacterial strains were grown in Difco 7H9 medium supplemented with 10% oleic acid-albumin-dextrose-catalase (OADC) and 5% glycerol. Lysine (80 μg/ml) and pantothenate (24 μg/ml) were added to mc^2^6020 medium. Tyloxapol was added to liquid cultures at a final concentration of 0.05%. Mycobactin J (Allied Monitor) was added to M. paratuberculosis cultures at a final concentration of 10 mg/liter. Bacteroides fragilis ATCC 25282D-5 and Lactobacillus reuteri ATCC 23272 were grown in brain heart infusion broth supplemented (per liter) with 5 g yeast extract, 10 ml 10% (wt/vol) l-cysteine HCl, 15 mg/liter hemin, and 66 ml 1.5 M 3-(*N*-morpholino)propanesulfonic acid [MOPS; pH 7.0] in an anaerobic chamber. Kanamycin was used at a final concentration of 50 mg/liter where appropriate.

### Isolation and cultivation of soil bacteria.

Soil samples (1 g) were vigorously agitated in 10 ml of deionized water for 10 min and were then allowed to sit for 10 min to allow large soil particles to settle. The supernatant was diluted into molten SMS medium ((0.125 g casein, 0.1 g potato starch, 1 g Casamino Acids, 20 g Bacto agar in 1 liter of water). Aliquots were then dispensed into the wells of 96-well microtiter plates or iChips (10). The microtiter plates were incubated at room temperature in humidified chambers for as long as 12 weeks, and the appearance of colonies was monitored weekly. At weekly intervals starting after 4 weeks, colonies were picked onto SMS medium. The iChips were placed in direct contact with the soil. After 4 weeks of incubation, the iChips were disassembled and the colonies picked onto SMS medium in order to test for the ability to propagate outside the iChip and to purify colonies. Glycerol stocks (15% glycerol) were made for isolates with robust growth on SMS medium.

### Extract preparation and screening.

Isolates from the NovoBiotic collection were transferred to seed broth (15 g glucose, 10 g malt extract, 10 g glycerol, 2.5 g yeast extract, 5 g Casamino Acids, and 0.2 g calcium carbonate chips·2H_2_O per liter of deionized H_2_O [pH 7.0]) and incubated with vigorous agitation at 28°C for 5 to 12 days until the culture was turbid (by visual inspection). The time to turbidity depends on the isolate. The seed culture was then diluted 1:20 into three different fermentation media. After 11 days of growth at 28°C with agitation, the cultures were dried down. Dimethyl sulfoxide (DMSO) was added to the dried biomass and mixed, and the crude extracts were tested for activity against S. aureus. A 5-μl aliquot of each extract was applied to a lawn of S. aureus growing on Mueller-Hinton agar. After overnight incubation at 37°C, the presence of a clearing zone indicated hit activity.

The extracts were tested against M. tuberculosis by transferring 1.5 μl of extracts to the wells of a 96-well microtiter plate. A culture of M. tuberculosis mc^2^6020 expressing mCherry was grown in Middlebrook 7H9 broth at 37°C with agitation to an optical density at 600 nm (OD_600_) of 0.4 to 0.5. The culture was diluted to an OD_600_ of 0.003 and 148.5 μl of culture added to the wells (1:100 dilution). After 7 days of incubation with agitation at 37°C, growth was monitored by measuring the OD_600_ and by measuring fluorescence with excitation at 580 nm and emission at 610 nm. Extracts that demonstrated ≤25% growth relative to that of the DMSO positive-growth controls were cherry picked and retested under the same assay conditions. These extracts were also tested at a 1:100 dilution in a broth microdilution assay against S. aureus. Extracts without any effect on the growth of S. aureus that repeated the inhibition of the growth of M. tuberculosis were considered confirmed hits.

Three strains were chosen for additional fermentation to provide material. IS019924, IS019923, and IS008612 are the producer strains for amycobactin, streptomycobactin, and kitamycobactin, respectively. Grown biomass of each isolate was inoculated into 50 ml seed broth and was grown with shaking at 200 rpm for 3 to 8 days. The cultures were monitored visually daily for robust growth. A 2% inoculum of the seed culture was transferred into 500 ml fermentation medium in 2-liter, 6-baffle flasks at 28°C, shaken at 200 rpm, and harvested after 7 days. IS019924 was fermented in BPM fermentation medium [20 g glucose, 10 g organic soy flour (Bob’s Red Mill organic whole ground soy flour), 10 g Pharmamedia (Traders protein), 1 g (NH_4_)_2_SO_4_, 10 g CaCO_3_, and 20 g glycerol in 1 liter] and required a total of 40 liters of production for the isolation of sufficient material to characterize amycobactin. IS019923 and IS008612 were fermented in R4 fermentation medium (described previously by Ling et al. [7]) and required 40 liters for the isolation of sufficient material.

### Isolation protocol for amycobactin.

The fermentation broth of IS019924 (10 liters) was centrifuged at 17,700 × *g* for 30 min. The supernatant was decanted and passed slowly over a column of HP20 resin. The HP20 resin was eluted using a step gradient (2 liters of 30% acetone in water, 2 liters of 80% acetone in water, and 2 liters of 100% acetone). The cell pellet was extracted with acetone and filtered. The acetone pellet extract was dried onto HP20 resin by rotary evaporation. The HP20 resin containing the pellet extract was washed with deionized water (1 liter) and eluted using a step gradient (1 liter of 20% acetone in water, 1 liter of 40% acetone in water, 1 liter of 60% acetone in water, 1 liter of 80% acetone in water, and 1 liter of 100% acetone). Amycobactin was determined to be present in the 80% fraction of the supernatant and in both the 60% and 80% fractions of the pellet extract. These fractions were combined, dried completely, and then reconstituted in methanol. This mixture was further separated on a column of LH20 resin (eluted in methanol), yielding 3 fractions containing amycobactin. These fractions were again combined and dried completely. The residue was dissolved in DMSO and was separated via HPLC (Agilent Zorbax SB-C_18_ column; particle size, 5 μm; inside diameter, 9.4 mm; length, 250 mm) (solvent A, H_2_O–0.1% trifluoroacetic acid [TFA]; solvent B, acetonitrile [ACN]–0.1% TFA; gradient, 10% B from 0 to 3 min and 10% to 100% B from 3 to 20 min; flow rate, 3.0 ml/min). Amycobactin eluted at 19 min, and a second purification via HPLC was performed (Agilent Zorbax SB-C_18_ column; particle size, 5 μm; inside diameter, 9.4 mm; length, 250 mm) (solvent A, H_2_O–0.1% TFA; solvent B, ACN–0.1% TFA; gradient, 55% B from 0 to 3 min and 55% to 100% B from 3 to 20 min; flow rate, 3.0 ml/min). Purified amycobactin eluted from the column at 10 min. Fractions containing amycobactin were lyophilized in preparation for structural analysis and biological testing.

### Isolation protocol for streptomycobactin.

The fermentation broth (8.5 liters) was centrifuged at 17,700 × *g* for 45 min. The cell pellet was extracted with 1 liter of acetone. The supernatant was extracted with 2 liters of *n*-butanol, and the aqueous layer was discarded. The acetone pellet extract and the *n*-butanol extract of the supernatant were added to separate round-bottom flasks, and HP20 was added to each flask. The organic solvents were removed via rotary evaporation. The HP20 resins were washed with deionized water and eluted using a step gradient (25% acetone in water, 50% acetone in water, and 100% acetone). Streptomycobactin was found in the 50% and 100% elutions. These elutions were then combined and dried, leaving a brown residue. Hexane was added to the residue, which was then sonicated and centrifuged. The hexane extract was then discarded, and the residue was dissolved in DMSO and separated via HPLC (Agilent Zorbax SB-C_18_ column; particle size, 5 μm; inside diameter, 9.4 mm; length, 250 mm) (solvent A, H_2_O–0.1% TFA; solvent B, ACN–0.1% TFA; gradient, 80% A to 55% B over 22 min; flow rate, 3.0 ml/min). The fractions containing streptomycobactin were combined and lyophilized to leave a white powder.

### Isolation protocol used for ^13^C- and ^15^N-labeled streptomycobactin.

Grown biomass of IS019923 was inoculated into 20 ml modified CM-R4 (10 g MgCl_2_·6H_2_O, 4 g CaCl_2_·2H_2_O, 0.2 g K_2_SO_4_, 5.6 g of 2-[{1,3-dihydroxy-2-(hydroxymethyl)propan-2-yl}amino]ethanesulfonic acid [TES free acid], 10 g of ^13^C-labeled glucose [U-^13^C_6_, 99%; CLM-1396-5; Cambridge Isotope Laboratories], 10 ml of 0.25-g/ml ^13^C-, ^15^N-labeled Celtone base powder [^13^C, 98%+; ^15^N, 98%+; CGM-1030P-CN-1; Cambridge Isotope Laboratories]) with the pH adjusted to 7.0. The culture was placed on a rotatory shaker at 200 rpm and 28°C for 7 days. The culture was monitored visually daily for robust growth that attached itself tightly to the sides of the flask at the liquid-air interface. A 2% inoculum of this culture was transferred into 500 ml of modified CM-R4 medium in a 2-liter, 6-baffle flask at 28°C, shaken at 200 rpm, and harvested after 9 days.

The fermentation broth (500 ml) was centrifuged at 17,700 × *g* for 45 min. The supernatant was decanted into empty centrifuge bottles, mixed with an equal volume of *n*-butanol, and then centrifuged. The cell pellet was extracted with 200 ml of acetone, sonicated for 10 min, and then centrifuged. The acetone pellet extract and the *n*-butanol supernatant extract were mixed together in a round-bottom flask, and HP20 was added. The organic solvents were removed via rotary evaporation. The HP20 resin was washed with deionized water and eluted using a step gradient (500 ml of 20% acetone in water, 500 ml of 50% acetone in water, and 500 ml of 100% acetone). Labeled streptomycobactin was found in the 50% and 100% elutions, which were dried separately, leaving brown residues. These residues were dissolved in DMSO, separated via HPLC (Agilent Zorbax SB-C_18_ column; particle size, 5 μm; inside diameter, 9.4 mm; length, 250 mm) (solvent A, H_2_O–0.1% TFA; solvent B, ACN–0.1% TFA; gradient, 90% A to 100% B over 22 min; flow rate, 3.0 ml/min). Fractions containing labeled streptomycobactin were combined and lyophilized to leave a white powder. The powder was dissolved in DMSO, and the sample was then repurified via HPLC (Agilent Zorbax SB-C_18_ column; particle size, 5 μm; inside diameter, 9.4 mm; length, 250 mm) (solvent A, H_2_O–0.1% TFA; solvent B, ACN–0.1% TFA; gradient, 80% A to 55% B over 22 min; flow rate, 3.0 ml/min). The fractions containing ^13^C- and ^15^N-labeled streptomycobactin were combined and lyophilized to leave 10.4 mg of a white powder.

### Isolation protocol used for kitamycobactin.

The fermentation broth was centrifuged at 17,700 × *g* for 45 min. The supernatant was decanted into a separatory funnel, and the remaining cell pellet was extracted with acetone before centrifugation. The acetone extract was transferred to a round-bottom flask, and the acetone was removed via rotary evaporation. The remaining water layer was added to the supernatant, and then the mixture was extracted with *n*-butanol. The water layer was discarded, and the *n*-butanol was transferred to a round-bottom flask. The *n*-butanol was removed via rotary evaporation. The remaining residue was dissolved in DMSO and then purified via HPLC (Agilent Zorbax SB-C_18_ column; particle size, 5 μm; inside diameter, 9.4 mm; length, 250 mm) (solvent A, H_2_O–0.1% TFA; solvent B, ACN–0.1% TFA; gradient, 90% A to 100% B over 22 min; flow rate, 3.0 ml/min). Kitamycobactin eluted at 24 min. The fraction was then lyophilized to leave a white powder.

### NMR.

A Bruker DRX 500-MHz spectrometer equipped with BBI and QNP probes was used to record the spectra of amycobactin and kitamycobactin. The samples were dissolved in dimethyl sulfoxide-*d*_6_ (Cambridge Isotope Laboratories, Andover, MA) and heated to 40°C for data acquisition. Structural assignments of amycobactin (Text S1) and kitamycobactin (Text S1) were made based on 1-dimensional (1D) and 2D nuclear magnetic resonance (NMR) data from ^1^H, ^13^C, COSY, TOCSY, ^1^H-^13^C/^15^N HSQC, ^1^H-^13^C HMBC, and NOESY/ROESY experiments (Text S1). A Bruker Avance III HD spectrometer operating at a ^1^H frequency of 700.13 MHz equipped with a cryogenically cooled triple-resonance 5-mm HCN probe was used to record the spectra of a sample of [^13^C, ^15^N]streptomycobactin dissolved in dimethyl sulfoxide-*d*_6_ (Cambridge Isotope Laboratories, Andover, MA) in a sample tube, and the sample was heated to 35°C for data collection. The structure of streptomycobactin was assigned based on a series of 1D, 2D, and 3D NMR experiments using the [^13^C, ^15^N]streptomycobactin sample (Text S1). For the peptidyl backbone ^13^C assignments, the HNCACB and HNCOCACB experiments were optimized for fast data acquisition using parameters published by P. Schanda et al. in 2006 (33). Side-chain assignments were mapped with the (H)CCCONH and H(CCCO)NH experiments for ^13^C and ^1^H assignments, respectively (Text S1).

### MIC.

MICs were determined by broth microdilution. Cell concentrations were adjusted to an OD_600_ of 0.003 in 7H9 medium supplemented with 10% OADC, 5% glycerol, and 0.05% tyloxapol. Pantothenate (24 μg/ml) and lysine (80 μg/ml) were added to H37Rv mc^2^6020 cultures. Plates were incubated at 37°C for either 7 days (M. tuberculosis, M. paratuberculosis, M. avium), 3 days (M. smegmatis, M. abscessus), or 20 h (B. fragilis, L. reuteri). B. fragilis and L. reuteri were grown under anaerobic conditions. The MIC was defined as the lowest concentration of antibiotic with no visible growth.

### Mammalian cytotoxicity.

Exponentially growing NIH/3T3 mouse embryonic fibroblasts (ATCC CRL-1658) in Dulbecco’s modified Eagle’s medium supplemented with 10% bovine calf serum and HepG2 cells (ATCC HB-8065) in Dulbecco’s modified Eagle’s medium supplemented with 10% fetal calf serum were seeded into 96-well flat-bottom plates. After 24 h of incubation at 37°C, the medium was replaced with fresh medium containing 2-fold serial dilutions of test compounds. After 72 h of incubation at 37°C, viability was determined with the CellTiter 96 AQueous One Solution cell proliferation assay kit (catalog no. G3580; Promega) according to the manufacturer’s recommendations.

### Time-dependent killing.

Exponential-phase cultures were prepared by growing M. tuberculosis to mid-exponential phase (OD_600_, ∼1 to 1.5) and then back diluting to an OD_600_ of 0.003. For stationary-phase cultures, M. tuberculosis was grown for 2 weeks to an OD_600_ of >1.5. Cultures were challenged with either 4× MIC (amycobactin) or 10× MIC (streptomycobactin, kitamycobactin, and marfomycin D) of the compound at 37°C. At intervals, 100-μl aliquots were removed, washed once in phosphate-buffered saline (PBS), serially diluted, and plated onto 7H10 medium to determine the CFU count per milliliter.

### Amycobactin mutant generation.

Mutants resistant to amycobactin in M. smegmatis were selected by plating onto 7H10 medium containing amycobactin at 10× wild-type MIC. Briefly, 10 ml of wild-type M. smegmatis was grown to an OD_600_ of ∼1.0. The culture was washed once and was concentrated to 1/10 the original volume (1 ml). The culture was then plated onto five 7H10 plates containing 10× MIC amycobactin. Mutants were streaked onto nonselective medium, and MICs were determined. Mutants were sent for whole-genome sequencing, and variant analysis was conducted by MR DNA. Targeted mutations were made in M. smegmatis and M. tuberculosis via single-stranded recombineering as in reference 19 with plating on 10× MIC amycobactin. The oligonucleotides used to make targeted mutations were 5′-CGAGACCGACACCGATCATGATCAGAACCGCGGTCGGCAGGTTCTGTACCGAACCGGTGTTCCCGATCTC-3′ for Msm*ΔsecY* 3AA, 5′-CCCTGCTCGACGAACACCACGCCGATGATGATCACGGCGGTGAACACGACGCCGCCGCGGCTCTCCAGGA-3′ for Msm*ΔsecY* 6AA, and 5′-CCAAACCGACACCGATCATGATCAGCACCGCGGTAGGCAGGTTCTGCACGGTTCCACCGGCGCCGATCTG-3′ for Mtb*ΔsecY* 3AA. Targeted mutations were confirmed via PCR and Sanger sequencing.

### β-Lactamase activity assay and Western blotting.

Duplicate 10-ml cultures of the M. smegmatis ′BlaTEM-1 reporter strain were grown in minimal medium with 0.02% glucose and 0.05% Tween 80 to late-exponential phase. The cultures were washed twice in minimal medium (34) and resuspended in 1 ml minimal medium with 0.02% glucose and without Tween 80. One culture was left untreated, and the other was treated with 10× MIC amycobactin. The cultures were incubated in a 37°C static incubator for 3 h with gentle agitation every 30 min. The bacteria were then pelleted, and the supernatant was carefully aspirated and filtered through a 0.45-μm filter (Costar). The filtrate was then concentrated 10-fold using a 3-kDa molecular-weight-cutoff spin column (Millipore). The pelleted bacteria were resuspended in PBS and subjected to bead beating to lyse the cells. Samples were spun at 12,000 rpm for 5 min, and the clarified lysate was aspirated. A Thermo Fisher bicinchoninic acid (BCA) kit was used to quantify protein yield. β-Lactamase activity was monitored by hydrolysis of nitrocefin. A total of 10 μg protein was diluted in a final volume of 100 μl in PBS and added to a 96-well plate. A 100-μl volume of the assay mixture was added containing 100 μg/ml nitrocefin, 200 μg/ml bovine serum albumin (BSA), and 10% glycerol. β-Lactamase activity was monitored by reading the absorbances at 490 nm and 390 nm every 5 min for 1 h. Western blot analysis was conducted by running 5 μg total protein from each sample on a Novex 4-to-20% Tris-glycine gel (Invitrogen), followed by transfer to a polyvinylidene difluoride (PVDF) membrane. An antibody against E. coli β-lactamase was purchased from QED Biosciences and was used at a 1:5,000 dilution in 5% milk; the mixture was incubated overnight. Densitometry was performed with ImageJ software.

### Protein structure depiction.

The PyMOL Molecular Graphics System, version 2.3.3 (Schrödinger), was used for protein depiction.

### Statistical analysis.

Analysis was performed with GraphPad Prism, version 7.
