# Supplementary figures and images for: Novel Antimicrobials from Uncultured Bacteria Acting against Mycobacterium tuberculosis
Source: mBio. 2020 Aug 4;11(4):e01516-20. doi: 10.1128/mBio.01516-20 (PMC7407088; doi:10.1128/mBio.01516-20)

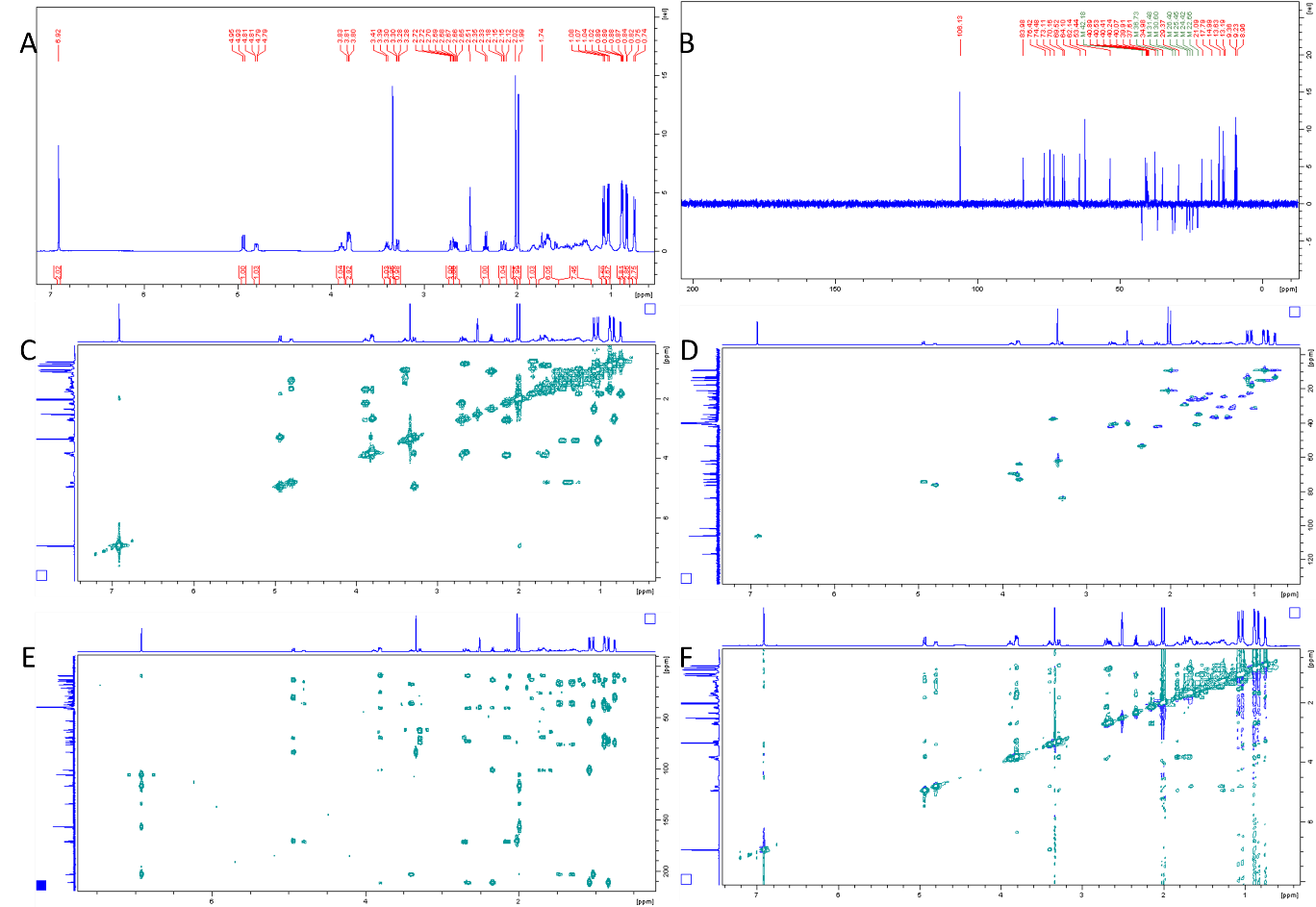

Supplement: FIG S2 [file mBio.01516-20-sf002.tif]

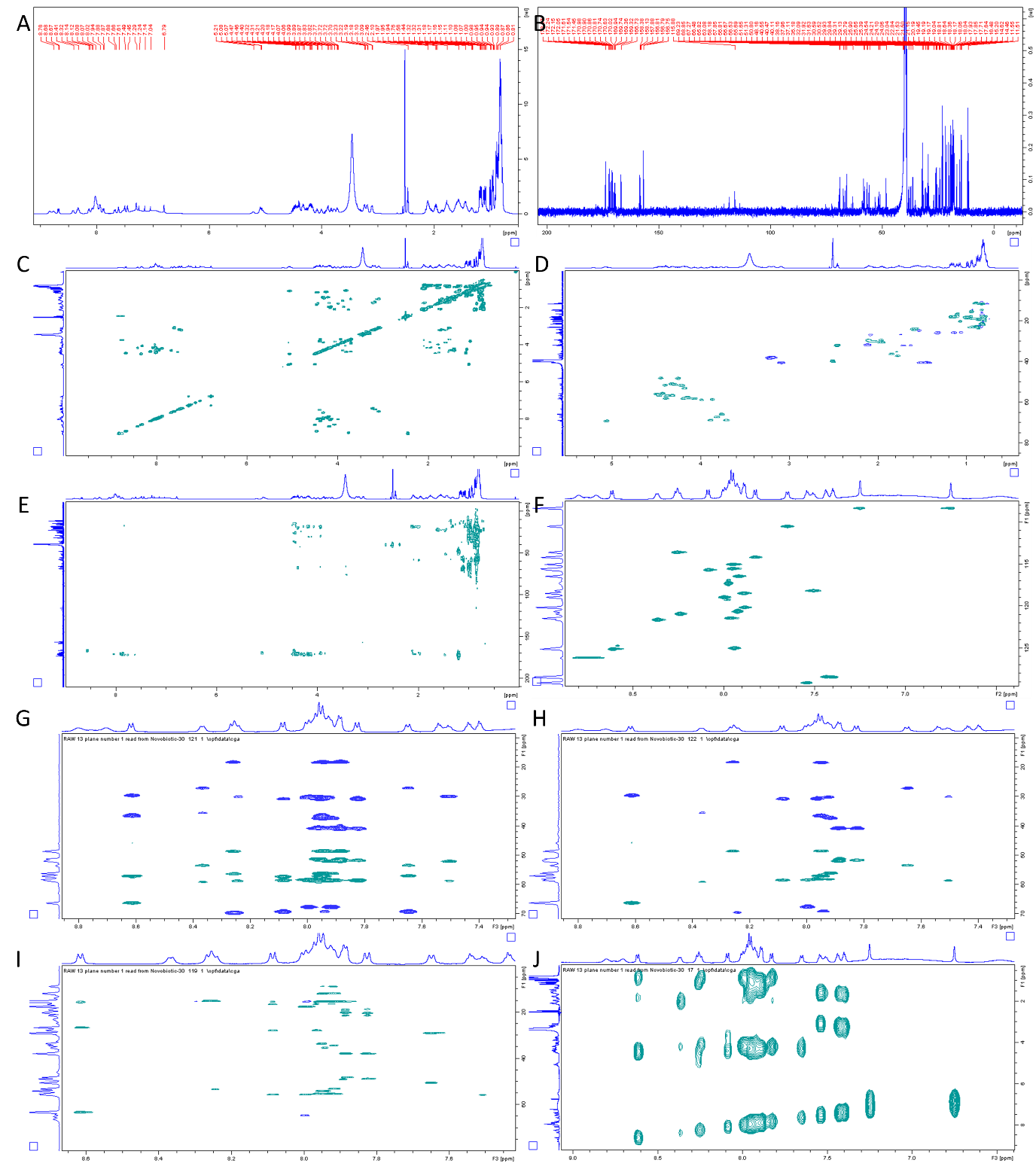

Supplement: FIG S3 [file mBio.01516-20-sf003.tif]

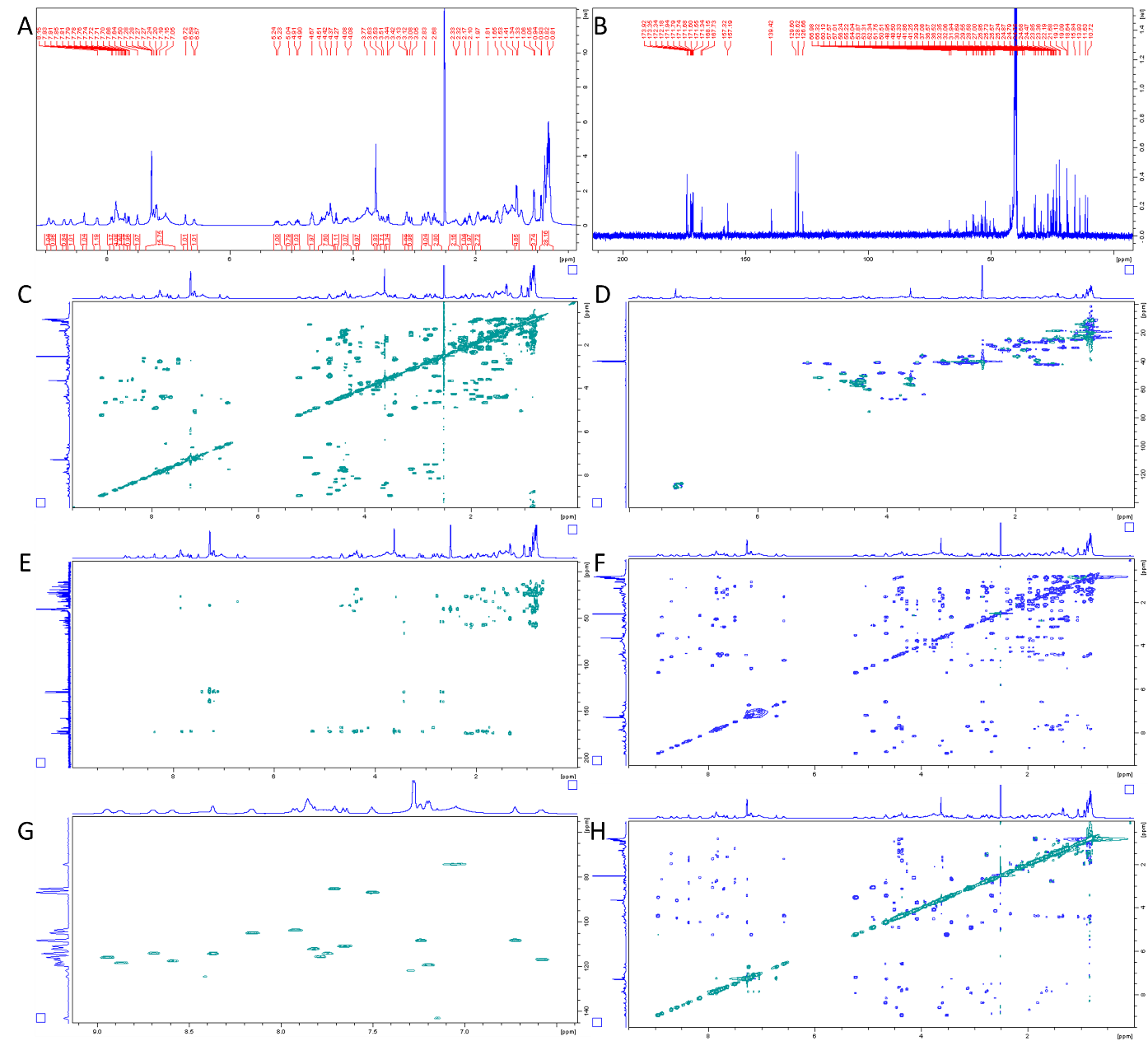

Supplement: FIG S4 [file mBio.01516-20-sf004.tif]
