# Supplementary material for: Novel Antimicrobials from Uncultured Bacteria Acting against Mycobacterium tuberculosis
Source: mBio. 2020 Aug 4;11(4):e01516-20. doi: 10.1128/mBio.01516-20 (PMC7407088; doi:10.1128/mBio.01516-20)
Supplement: TABLE S1 [file mBio.01516-20-st001.docx]

**Table S1. ^1^H and ^13^C NMR data of amycobactin (500/125 MHz in DMSO-*d_6_*, δ in ppm)**

| Position | C, type | H, mult. (*J* in Hz)^a^ | COSY^b^ | HMBC^b^ |
| --- | --- | --- | --- | --- |
| 1 | 171.6, C | - | - | - |
| 2 | 42.1, CH_2_ | 2.14/2.70, dd (2.5, 14.3) | 3 | 1, 3, 4 |
| 3 | 69.4, CH | 3.88, dt (2.5, 10.0) | 2, 4 | - |
| 4 | 40.8, CH | 1.68, m | 3, 5, 30 | 3, 6, 30 |
| 5 | 73.1, CH | 3.79, m | 4, 6 | 3, 4, 6, 7, 30, 31 |
| 6 | 40.5, CH | 2.66, m | 5, 31 | 4, 5, 31 |
| 7 | 211.5, C | - | - | - |
| 8 | 53.4, CH | 2.34, q (7.3) | 32 | 6, 7, 9, 32 |
| 9 | 101.6, C | - | - | - |
| 10 | 25.3, CH_2_ | 1.58/1.71, m | - | 9, 11, 12 |
| 11 | 26.3, CH_2_ | 1.66/1.74, m | 12 | 9, 13 |
| 12 | 64.0, CH | 3.80, m | 11 | 10 |
| 13 | 70.1, CH | 3.82, m | 14 | 12 |
| 14 | 83.9, CH | 3.28, dd (10.4, 2.4) | 13, 15 | 12, 13, 15, 33 |
| 15 | 74.4, CH | 4.93, app. d (10.4) | 14, 16 | 13, 14, 16, 17, 34, 36 |
| 16 | 29.3, CH | 1.82, m | 15, 17, 36 | 17, 36 |
| 17 | 31.4, CH_2_ | 1.00/1.26, m | 16 | - |
| 18 | 24.4, CH_2_ | 1.14/1.35, m | - | - |
| 19 | 22.5, CH_2_ | 1.04/1.53, m | - | - |
| 20 | 30.5, CH_2_ | 1.26/1.40, m | 19, 21 | - |
| 21 | 76.4, CH | 4.79, app. d (10.5) | 20, 22 | 1, 37 |
| 22 | 34.9, CH | 1.66, m | 21, 23, 37 | - |
| 23 | 36.7, CH_2_ | 1.30/1.46, m | 22, 24 | 21, 22, 24, 25, 37, 38 |
| 24 | 37.5, CH | 3.39, m | 23, 38 | 22, 23, 25, 38 |
| 25 | 203.4, C | - | - | - |
| 26 | 134.5, C | - | - | - |
| 27/27’ | 106.0, CH | 6.91, s | - | 25, 26, 28/28’, 29 |
| 28/28’ | 156.9, C | - | - | - |
| 29 | 116.8, C | - | - | - |
| 30 | 9.0, CH_3_ | 0.87, d (7.0) | 4 | 3, 4, 5 |
| 31 | 9.1, CH_3_ | 0.83, d (6.5) | - | 5, 7 |
| 32 | 13.6, CH_3_ | 1.07, d (7.3) | 8 | 7, 8, 9 |
| 33 | 62.1, CH_3_ | 3.33, s | - | 14 |
| 34 | 170.6, C | - | - | - |
| 35 | 21.0, CH_3_ | 2.02, s | - | 34 |
| 36 | 13.1, CH_3_ | 0.74, d (6.5) | 16 | 15, 16, 17 |
| 37 | 14.9, CH_3_ | 0.89, d (6.7) | 22 | 21, 22, 23 |
| 38 | 17.7, CH_3_ | 1.02, d (6.8) | 24 | 23, 24, 25 |
| 39 | 9.1, CH_3_ | 1.99, s | - | 26, 27/27’, 28/28’, 29 |

^a^ Proton multiplicity is shown as singlet (s), doublet (d), triplet (t), quartet (q), and the apparent pattern (app.).

^b^ COSY and HMBC correlations are from proton(s) stated to the indicated proton and carbon, respectively.
