# Supplementary material for: Novel Antimicrobials from Uncultured Bacteria Acting against Mycobacterium tuberculosis
Source: mBio. 2020 Aug 4;11(4):e01516-20. doi: 10.1128/mBio.01516-20 (PMC7407088; doi:10.1128/mBio.01516-20)
Supplement: TABLE S2 [file mBio.01516-20-st002.docx]

**Table S2: ^1^H, ^13^C and ^15^N NMR data of streptomycobactin (700/175/70 MHz in DMSO-*d_6_*, δ in ppm)**

| Position | HN | Hα | Hβ | Others |
| --- | --- | --- | --- | --- |
| Val 1 | 8.71/8.80 | 3.76 | 2.11 | Hγ1: 0.95, Hγ2: 0.99, Hδ(N-CH_3_): 2.46 |
| Ile 2 | 8.61 | 4.46 | 1.85 | Hγ1: 1.14/1.33, Hγ2: 0.83, Hδ: 0.85 |
| Ile 3 | 7.95 | 4.43 | 1.78 | Hγ1: 1.06/1.33, Hγ2: 0.79, Hδ: 0.82 |
| Thr 4 | 7.92 | 4.39 | 3.82 | Hγ1(CH_3_): 1.08, Hγ2(OH): 5.12 |
| Val 5 | 8.00 | 4.17 | 2.05 | Hγ1: 0.84, Hγ2: 0.84 |
| Leu 6 | 7.93 | 4.23 | 1.44 | Hγ: 1.58, Hδ1: 0.82, Hδ2: 0.88 |
| Leu 7 | 7.89 | 4.32 | 1.44 | Hγ: 1.60, Hδ1: 0.82, Hδ2: 0.86 |
| Val 8 | 7.83 | 4.19 | 1.98 | Hγ1: 0.82, Hγ2: 0.82 |
| β-OH Arg 9 | 8.08 | 4.36 | 3.72 | Hγ: 1.53/1.71, Hδ: 3.18, Hε: 7.44 |
| Ala 10 | 7.94 | 4.44 | 1.18 | - |
| Val 11 | 7.95 | 4.14 | 1.96 | Hγ1: 0.82, Hγ2: 0.82 |
| Leu 12 | 7.96 | 4.3 | 1.49 | Hγ: 1.58, Hδ1: 0.82, Hδ2: 0.88 |
| Ala 13 | 7.88 | 4.26 | 1.16 | - |
| Thr 14 | 8.26 | 4.5 | 5.07 | Hγ: 1.11 |
| Val 15 | 8.24 | 4.06 | 2.09 | Hγ1: 0.79, Hγ2: 0.79 |
| Arg 16 | 7.51 | 4.39 | 1.60/1.76 | Hγ: 1.54, Hδ: 3.09, Hε: 7.54 |
| Ile 17 | 7.98 | 4.01 | 1.8 | Hγ1: 1.16/1.40, Hγ2: 0.94, Hδ: 0.89 |
| Gln 18 | 8.37 | 4.19 | 1.75/2.08 | Hγ: 2.11, Hε: 6.75/7.25 |
| β-OH Arg 19 | 7.65 | 4.37 | 3.89 | Hγ: 1.63/1.71, Hδ: 3.18, Hε: 7.398 |
| Val 20 | 7.98 | 3.89 | 1.97 | Hγ1: 0.84, Hγ2: 0.84 |
|  |  |  |  |  |
| Position | N | Cα | Cβ | Others |
| Val 1 | 126.1 | 66.4 | 29.9 | Cγ1: 18.4, Cγ2: 18.8, Cδ(N-CH_3_): 32.4 |
| Ile 2 | 125.0 | 57.1 | 36.8 | Cγ1: 26.2, Cγ2: 15.0, Cδ: 12.0 |
| Ile 3 | 115.5 | 56.1 | 37.4 | Cγ1: 26.1, Cγ2: 14.9, Cδ: 12.0 |
| Thr 4 | 116.4 | 58.6 | 67.8 | Cγ1: 20.3 |
| Val 5 | 118.9 | 58.3 | 30.4 | Cγ1: 18.3, Cγ2: 19.6 |
| Leu 6 | 120.6 | 52.1 | 41.1 | Cγ: 24.6, Cδ1: 22.1, Cδ2: 23.3 |
| Leu 7 | 118.4 | 51.4 | 41.0 | Cγ: 24.6, Cδ1: 22.1, Cδ2: 23.3 |
| Val 8 | 114.1 | 58.6 | 31.0 | Cγ1: 18.6, Cγ2: 19.7 |
| β-OH Arg 9 | 115.6 | 57.4 | 69.3 | Cγ: 32.6, Cδ: 38.4, Nε: 128.3 |
| Ala 10 | 124.9 | 48.9 | 18.5 | - |
| Val 11 | 115.0 | 58.3 | 30.8 | Cγ1: 18.6, Cγ2: 19.6 |
| Leu 12 | 121.4 | 51.8 | 41.1 | Cγ: 24.6, Hδ1: 21.8, Cδ2: 23.3 |
| Ala 13 | 120.1 | 48.9 | 18.3 | - |
| Thr 14 | 113.5 | 56.4 | 69.6 | Cγ: 17.1 |
| Val 15 | 120.9 | 58.9 | 30.2 | Cγ1: 18.2, Cγ2: 19.8 |
| Arg 16 | 118.1 | 52.1 | 30.0 | Cγ: 25.1, Cδ: 41.0, Nε: 129.0 |
| Ile 17 | 117.2 | 59.3 | 35.6 | Cγ1: 25.8, Cγ2: 15.7, Cδ: 11.8 |
| Gln 18 | 121.6 | 53.6 | 27.4 | Cγ: 32.1, Nε: 108.3 |
| β-OH Arg 19 | 110.4 | 57.4 | 69.4 | Cγ: 32.4, Cδ: 38.5, Nε: 128.4 |
| Val 20 | 119.2 | 59.0 | 30.1 | Cγ1: 18.3, Cγ2: 19.6 |
