# Supplementary material for: Novel Antimicrobials from Uncultured Bacteria Acting against Mycobacterium tuberculosis
Source: mBio. 2020 Aug 4;11(4):e01516-20. doi: 10.1128/mBio.01516-20 (PMC7407088; doi:10.1128/mBio.01516-20)
Supplement: TABLE S3 [file mBio.01516-20-st003.docx]

**Table S3. ^1^H, ^13^C, and ^15^N NMR data of kitamycobactin (500/125/50 MHz in DMSO-*d_6_*, δ in ppm)**

| Position | HN | Hα | Hβ | Others |
| --- | --- | --- | --- | --- |
| Gly 1 | 7.92 | 3.07/4.92 | - | - |
| Phe 2 | 8.87 | 4.66 | 2.67/3.42 | Hδ: 7.27, Hε: 7.28, Hζ: 7.20 |
| Gly 3 | 8.15 | 2.60/4.14 | - | - |
| Arg 4 | 7.74 | 4.36 | 1.52/1.79 | Hγ: 1.44/1.55, Hδ: 3.12, Hε: 7.70 |
| Ile 5 | 7.78 | 4.46 | 1.65 | Hγ1: N.D.^a^, Hγ2: 0.87, Hδ: N.D.^a^ |
| Lys 6 | 8.59 | 3.66 | 1.95/2.38 | Hγ: 1.36, Hδ: 1.54, Hε: 2.77, Hζ: 7.85 |
| Ala 7 | 8.41 | 4.35 | 1.33 | - |
| Asp 8 | 6.58 | 4.67 | 1.74/2.84 | - |
| Gln 9 | 8.37 | 3.59 | 1.67/1.81 | Hγ: 2.11/2.17, Hε: 6.72/7.23 |
| Leu 10 | 7.64 | 4.41 | 1.40/1.64 | Hγ: 1.43, Hδ1: 0.88, Hδ2: 0.83 |
| Val 11 | 7.82 | 4.37 | 1.86 | Hγ1: 0.82, Hγ2: 0.82 |
| Gly 12 | 8.94 | 3.52/5.24 | - | - |
| Arg 13 | 7.19 | 5.04 | 1.05/1.24 | Hγ: 1.26, Hδ: 2.67/2.84, Hε: 7.50 |
| Leu 14 | 7.28 | 4.42 | 1.34/1.48 | Hγ: 1.45, Hδ1: 0.82, Hδ2: 1.05 |
| Ile 15 | 8.69 | 4.5 | 1.96 | Hγ1: 0.96/1.08, Hγ2: 0.93, Hδ: 0.78 |
| Pro 16 | - | 4.27 | 1.97/2.32 | Hγ: 1.89/2.09, Hδ: 3.76/4.08, Hε(O-CH_3_): 3.62 |
|  |  |  |  |  |
| Position | N | Cα | Cβ | Others |
| Gly 1 | 103.3 | 41.7 | - | - |
| Phe 2 | 117.9 | 54.0 | 36.4 | Cγ:139.5, Cδ: 129.6, Cε: 128.5, Cζ: 126.5 |
| Gly 3 | 104.5 | 41.0 | - | - |
| Arg 4 | 113.7 | 53.7 | 31.8 | Cγ: 25.5, Cδ: 40.6, Nε: 84.8 |
| Ile 5 | 115.1 | 57.3 | 39.4 | Cγ1: N.D.^a^, Cγ2: 15.56, Cδ: N.D.^a^ |
| Lys 6 | 117.1 | 56.9 | 28.4 | Cγ: 24.37, Cδ: 26.85, Cε: 39.19, Nζ: 33.30 |
| Ala 7 | 123.9 | 50.5 | 18.6 | - |
| Asp 8 | 116.5 | 48.9 | 37.6 | Cγ: N.D.^a^ |
| Gln 9 | 113.7 | 56.9 | 26.9 | Cγ: 32.02, Cδ: N.D.^a^, Nε: 107.88 |
| Leu 10 | 110.5 | 55.3 | 42.3 | Cγ: 24.57, Cδ1: 23.26, Cδ2:23.81 |
| Val 11 | 111.7 | 57.5 | 32.2 | Cγ1: 19.20, Cγ2: 19.20 |
| Gly 12 | 115.3 | 41.3 | - | - |
| Arg 13 | 118.9 | 51.6 | 30.6 | Cγ: 25.66, Cδ: 41.15, Nε: 86.43 |
| Leu 14 | 121.4 | 53.3 | 42.5 | Cγ: 24.50, Cδ1: 22.12, Cδ2: 21.65 |
| Ile 15 | 113.6 | 55.1 | 36.6 | Cγ1: 24.74, Hγ2: 13.72, Cδ: 10.59 |
| Pro 16 | N.D.^b^ | 60.0 | 29.5 | Cγ: 25.26, Cδ: 48.46, Cε(O-CH_3_): 52.29 |

^a^ Not determined due to signal overlap.

^b^ Not determined with current NMR experiments
