# Supplementary material for: Novel Antimicrobials from Uncultured Bacteria Acting against Mycobacterium tuberculosis
Source: mBio. 2020 Aug 4;11(4):e01516-20. doi: 10.1128/mBio.01516-20 (PMC7407088; doi:10.1128/mBio.01516-20)
Supplement: TEXT S1 [file mBio.01516-20-s0001.docx]

**Structure elucidation of amycobactin**

Mass spectrometry analysis of amycobactin showed a protonated ion *m/z* of 763 [M+H]^+^, indicating a molecular formula of C_41_H_62_O_13_ with 11 degrees of unsaturation. The structure was elucidated using ^1^H, ^13^C DEPT135, ^1^H-^1^H COSY, ^1^H-^13^C HSQC and ^1^H-^13^C HMBC experiments. A singlet proton signal H-27/27’ (δ_H_ 6.87) was integrated to two protons with HSQC correlations to C-27/27’ (δ_C_ 106.0), suggesting a symmetry in an aromatic system. The HMBC correlations from H-27/27’ to C-26 (δ_C_ 134.5), C-28/28’ (δ_C_ 156.9), and C-29(δ_C_ 116.8) revealed the non-protonated carbons in the aromatic system. A singlet methyl signal H-39 (δ_H_ 1.99) integrated to three protons showed HMBC correlations to all carbons in the aromatic ring, indicating a methyl substitution located on a symmetry plane. An HMBC correlation from H-27/27’ to C-25 (δ_C_ 203.4) suggested a carbonyl substitution on the symmetry plane located opposite to the methyl substitution. A lack of HMBC correlation from H-27/27’ to C-39 supported the methyl substitution was located at the *meta*-position to H-27/27’. Dihydroxy substitutions at the aromatic ring was assigned based on the carbon chemical shifts at C28/28’. The polyketide backbone of the molecule was mainly elucidated by COSY experiments. HMBC correlations from the methylene protons H-23 (δ_H_ 1.30/1.46), the methine proton H-24 (δ_H_ 3.39), and the methyl protons H-38 (δ_H_ 1.02) to the carbonyl carbon C-25 established the connectivity between C-24 and C-25. It suggested one end of the polyketide backbone was connected to the aromatic ring via a carbonyl. The other end of the polyketide was cyclized as an ester moiety, as evidenced by an HMBC correlation from the methine proton H-21 (δ_H_ 4.79) and a methylene proton H-2 (δ_H_ 3.14/2.70) to the carbonyl carbon C-1 (δ_C_ 171.6). The macrolide ring contains two six-member heterocyclic rings connected through a ketal carbon. The first ring was established by HMBC correlations from the methine protons H-5 (δ_H_ 3.79) and H-8 (δ_H_ 2.34) to the carbonyl C-7 (δ_C_ 211.5), and H-8 also had HMBC correlations to C-6 (δ_C_ 40.5) and C-9 (δ_H_ 101.6). The COSY correlations between H-5 and methyl protons H-31 (δ_H_ 0.83), between H-8 and methyl protons H-32 (δ_H_ 1.07) suggested the C-5 and C-8 positions were methylated. In the second six-member ring, the H-12 (δ_H_ 3.80) and H-11 (δ_H_ 1.66/1.74) were connected with a COSY correlation; due to signal overlap, the COSY correlations between H-12 and H-13 (δ_H_ 3.82), and between H-10 (δ_H_ 1.58/1.71) and H-11 were unable to assign unambiguously. However, the HMBC correlations from H-10 and H-11 to C-9 (δ_C_ 101.6), and from H-10 to C-11 (δ_C_ 26.3) and C-12 (δ_C_ 64.0) were able to establish connectivity between C-9 and C-10 and between C-10 and C-11. Also, the ^3^*J*_H-C_ HMBC correlations from H-11 to C-13 (δ_C_ 70.1) and from H-14 (δ_H_ 3.28) to C-12 were able to connect C-12 and C-13. The relatively downfield carbon chemical shifts of C-5 (δ_C_ 73.1), C-9 (δ_H_ 101.6), and C-13 (δ_H_ 70.1), as well as the molecular formula requirement supported the double oxane ring assignment. Additionally, a NOE was able to establish the spatial proximity between H-8 and H-10, further supporting the double ring structure. A methoxy group was assigned at C-12 as evidenced by a ^3^*J*_H-C_ HMBC correlation from H-33 (δ_H_ 3.33) to C-14 (δ_C_ 83.9). An acetylation was assigned at the C-15 (δ_C_ 74.4) position based on HMBC correlations from H-15 (δ_H_ 4.93) and H-35 (δ_H_ 2.02) to C-34 (δ_C_ 170.6). Two oxygen atoms remained unassigned in the molecular formula, and the carbon chemical shits at C-3 (δ_C_ 69.4) and C-12 (δ_C_ 64.0) supported they were adjacent to oxygen atoms. Lastly, COSY correlation was unable to established between the two open-end methylene protons H-18 (δ_H_ 1.14/1.35) and H-19 (δ_H_ 1.04/1.53) due to signal overlap, the proton and carbon chemical shifts at these positions eliminated possibility of being adjacent to heteroatoms, therefore C-18 and C-19 must be connected by a single bond.
