# Supplementary material for: Novel Antimicrobials from Uncultured Bacteria Acting against Mycobacterium tuberculosis
Source: mBio. 2020 Aug 4;11(4):e01516-20. doi: 10.1128/mBio.01516-20 (PMC7407088; doi:10.1128/mBio.01516-20)
Supplement: TEXT S2 [file mBio.01516-20-s0002.docx]

**Structure elucidation of streptomycobactin**

Mass spectrometry analysis of the unlabeled streptomycobactin showed a protonated ion *m/z* of 2260 [M+H]^+^, indicating a molecular formula of C_104_H_190_N_30_O_25_ with 25 degrees of unsaturation. Structure elucidation was conducted using ^1^H, ^13^C, ^1^H-^1^H COSY, ^1^H-^13^C HSQC, ^1^H-^13^C HMBC experiments on the unlabeled streptomycobactin, and ^1^H-^15^N HSQC, HNCACB, HN(CO)CACB, CCCONH, ^15^N TOCSY-HSQC, ^13^C and ^15^N decoupled 2D NOESY and TOCSY experiments on the [^13^C, ^15^N]streptomycobactin sample. All amide protons were identified from the ^1^H-^15^N HSQC experiments. Using the HN(CO)CACB experiment, the α and β carbon chemical shifts of the amino acid preceding the amide NH group were identified. The identification was further confirmed by the HNCACB experiment, where α and β carbon chemical shifts from the same and preceding amino acid relative to the NH group were mapped. Using the CCCONH experiment, all carbon chemical shifts from the preceding amino acid residue were mapped using the amide proton. The ^15^N TOCSY-HSQC along with 2D NOESY and TOCSY experiments were used to identify the proton chemical shifts within each amino acid residue. The primary amide protons Gln18-Hε (δ_H_ 6.75/7.25) were assigned based on 2D NOESY correlations to Gln18-Hγ (δ_H_ 2.11). A methylation was assigned on the N-terminal of Val1 as evidenced by a ^1^H-^1^H COSY correlation between the N-methyl protons (δ_H_ 2.46) and the Val1-NH (δ_H_ 8.80). The C-terminal of the peptide was cyclized with the Thr14 oxygen forming an ester moiety, as suggested by the relatively downfield proton chemical shift of Thr14-Hβ (δ_H_ 5.07) and 2D NOESY correlations between Thr14-Hβ and Val20-Hα (δ_H_ 3.89). Additionally, both Thr14-Hβ and Val20-Hβ shared ^3^*J*_H-C_ HMBC correlations to the carbonyl carbon of Val20 (δ_C_ 170.2), further confirming the ester moiety. The Arg9-Cβ (δ_C_ 69.3) and Arg19-Cβ (δ_C_ 69.4) were methine carbons suggested in the phase-sensitive ^1^H-^13^C HSQC experiments, and their relatively downfield chemical shifts suggested they were both attached to a hydroxyl group.
