# Supplementary material for: Novel Antimicrobials from Uncultured Bacteria Acting against Mycobacterium tuberculosis
Source: mBio. 2020 Aug 4;11(4):e01516-20. doi: 10.1128/mBio.01516-20 (PMC7407088; doi:10.1128/mBio.01516-20)
Supplement: TEXT S3 [file mBio.01516-20-s0003.docx]

**Structure elucidation of kitamycobactin**

Mass spectrometry analysis of kitamycobactin showed a protonated ion *m/z* of 1736 [M+H]^+^, indicating a molecular formula of C_80_H_134_N_24_O_19_ with 26 degrees of unsaturation. Structure elucidation was conducted using ^1^H, ^13^C, ^1^H-^1^H COSY, ^1^H-^13^C HSQC, ^1^H-^13^C HMBC, ^1^H-^1^H TOCSY, ^1^H-^15^N HSQC, and 2D ROESY experiments. The amino acids spin systems were identified through COSY and TOCSY experiments. After the proton chemical shifts were identified from each amino acid, ^1^H-^13^C HSQC correlations were used to identify carbon chemical shifts. Two spins systems were identified for Phe2 and key ^1^H-^13^C HMBC correlations from Phe2-Hβ (δ_H_ 2.67) to Phe2-Cγ (δ_C_ 139.5) and Phe2-Cδ (δ_C_ 129.6) established connectivity of the two spin systems and supported a phenylalanine moiety. Following identification of individual spin systems, the amino acid sequence was mapped by the 2D ROESY experiment. The N-terminal of Gly1 was condensed with Asp8 forming an amide moiety, as evidenced by 2D ROESY correlations between Gly1-NH (δ_H_ 7.92) and Asp8-Hβ (δ_H_ 1.74/2.84). The C-terminal of Pro16 was methylated forming a methyl ester, supported by a ^3^*J*_H-C_ HMBC correlation from the methyl protons (δ_H_ 3.62) to the Pro16 carbonyl (δ_C_ 171.6) and a 2D ROESY correlation from the methyl protons to Pro16-Hα (δ_H_ 4.27).
